# Research Progress on Signaling Pathway-Associated Oxidative Stress in Endothelial Cells

**DOI:** 10.1155/2017/7156941

**Published:** 2017-04-19

**Authors:** Ying Liang, Jiajia Li, Qinlu Lin, Ping Huang, Lin Zhang, Wei Wu, Youchu Ma

**Affiliations:** ^1^National Engineering Laboratory for Rice and Byproduct Deep Processing, Central South University of Forestry and Technology, Changsha, Hunan 410004, China; ^2^College of Food Science and Engineering, Central South University of Forestry and Technology, Changsha, Hunan 410004, China

## Abstract

Studying the mechanisms of oxidative stress in endothelial cells is vital to the discovery of novel drugs for the treatment of cardiovascular disease. This article reviews the progress within the field of the role of oxidative responses in the physiology and growth of endothelial cells and emphasizes the effects of several main signal pathways involved in the oxidative stress of endothelial cells. Herein, we aim to provide scientific direction that can serve as a basis for researchers specializing in the signaling pathway of oxidative stress.

## 1. Introduction

An ever-increasing body of evidence supports the conclusion that many diseases, including hypertension, diabetes, atherosclerosis, and skin cancer, originate from endothelial oxidative stress. A range of factors can induce oxidative stress in endothelial cells. The consumption of high-fat food can lead to the production of considerable amounts of free fatty acids and contribute to damage and apoptosis of endothelial cells [1]. Furthermore, high concentrations of natural antioxidants, such as resveratrol, also affect mitochondrial membranes resulting in depolarization and inducing endothelial cells death [2]. Elucidating the various signal pathways involved in oxidative stress of endothelial cells may help facilitate the discovery of therapeutic targets for the treatment of diseases induced by endothelial oxidative stress.

## 2. Source of Endothelial Cells

Using cells as tools to study the biochemical and molecular mechanism of many cardiovascular and cerebrovascular diseases is essential. The sources of endothelial cells utilized in investigations can be classified by the species and organs from which they have been derived. The most commonly utilized endothelial cells are obtained from humans. Some animals are also sources of such cells used to establish experimental models, including cattle, swine, and rats (e.g., rat microvascular endothelial cells, RMVECs) [3]. Each experiment necessitates the use of specific cells from the corresponding organs, and, for example, human umbilical vein endothelial cells (HUVECs), which are obtained from human umbilical vein, are widely used and recognized by researchers as a well-established model. To study diseases associated with the blood-brain barrier, brain endothelial cells, such as human brain microvascular endothelial cell (HBMEC), are usually employed. In addition, experiments with primary human aortic endothelial cells (HAECs) are conducted to investigate hypertension-associated endothelial damage [4].

The aforementioned cell sources are primary in nature; however, utilization of an endothelial cell line, particularly to study the effects of carcinogens, is highly preferred. Thus, to study the relationship between oxidative stress and cancer, a relevant cell line needs to be obtained. In an earlier investigation, a HBMEC cell line [5] was used to explore the role of cigarette smoke in endothelial cells. Radiotherapy is often preferred as an important treatment modality for a large number of tumor types. For example, to examine the protective effect of paeoniflorin on endothelial cells against the adverse influence of gamma radiation used in radiotherapy, EA.hy926 [6], an immortalized cell line derived from HUVECs, which possesses the highly differentiated function of human umbilical vascular endothelium, was used.

## 3. The Role of Oxidative Stress in Endothelial Cells in Physiology and Growth

Oxidation in endothelial cells plays a key role in endothelium damage and is considered the common early mechanism contributing to the development of many cardiovascular diseases. The quantity of reactive species, including reactive oxygen species (ROS) and reactive nitrogen species (RNS) [7], increases when oxidation occurs in endothelial cells. The accumulation of ROS results in mitochondrial dysfunction, including the oxidation of lipids inside the mitochondria and a decrease of the mitochondrial transmembrane potential. The decline in the level of antioxidant enzymes may hinder the antioxidative capacity of the cell, leading to a series of self-perpetuated damage. Furthermore, oxidation may cause a decrease in the amount of nitric oxide (NO) and thus induce dysfunction of the endothelium [8]. Indeed, in a recent study, oxidative stress was found to promote apoptosis and result in the death of endothelial cells [9]. Endothelial cells participate and play an important role in the prevention of inflammation, regulate vascular tone, and maintain the balance between coagulation and anticoagulation. It has also been demonstrated that damage, apoptosis, and death of endothelial cells have been implicated in the development of diabetes mellitus [10], atherosclerosis [11], and other vascular diseases.

## 4. The Oxidation Processes in Endothelial Cell

Hydrogen peroxide (H_2_O_2_), oxidized low-density lipoprotein (ox-LDL), homocysteine (HCY), and advanced glycation end-products (AGE) generated in cells affect the redox potential within endothelial cells, when cells are stimulated by detrimental substances. Such agents exert pathophysiological effects on cells through oxidative stress when present in sustained elevated concentrations in plasma or tissues. Furthermore, extrinsic factors, such as environmental pollution, UV light, radiation, or the intake of high quantities of certain chemicals (e.g., glucose and fat or nanomaterials), can induce endothelium dysfunction. These factors lead to an imbalance in the redox potential of cells by inducing the production of oxidants and thereby reducing the level of antioxidants [12]. Irrespective of the stimulus, such processes are implicated in the oxidation of endothelial cells through certain signal pathways by a variety of regulatory factors, such as NF-*κ*B, Nrf2, and ARE.

## 5. Signaling Pathways Involved in Oxidative Stress

Under oxidative stress, the balance between oxidants and antioxidants in endothelial cells is disrupted. These results in the activation of the Nrf2/ARE pathway, which regulates the expression of antioxidative enzymes, for example, HO-1, leading to a restoration of the redox state balance. NF-*κ*B signaling is also activated and regulates cellular proliferation and apoptosis in inflammatory states. In addition, PI3K/AKT regulates vascular tone through the production of NO by modulating phosphorylation of eNOS after high glucose intake, resulting in endothelial cell injury. The MAPK family includes p38 MAPK, ERK1/2, and JNK [13]. ERK1/2 regulates the expression of NOX4 and subsequently modulates the production of ROS. The activation of JNK, and its substrate c-Jun, induces the expression of ROS which acts as an important secondary messenger and modulator for the ERK1/2/NOX4/ROS and JNK/c-Jun pathway [14]. P38MAPK also functions to control proliferation, differentiation, and migration of endothelial cells. A recent study revealed that as a newly discovered partner of p38 in HUVECs, nucleophosmin (NPM) impairs DNA damage response [15].

## 6. The Role of Each Specific Pathway Associated with Oxidative Stress in Endothelial Cells

### 6.1. Nrf2/ARE Pathway

**Figure 1:**
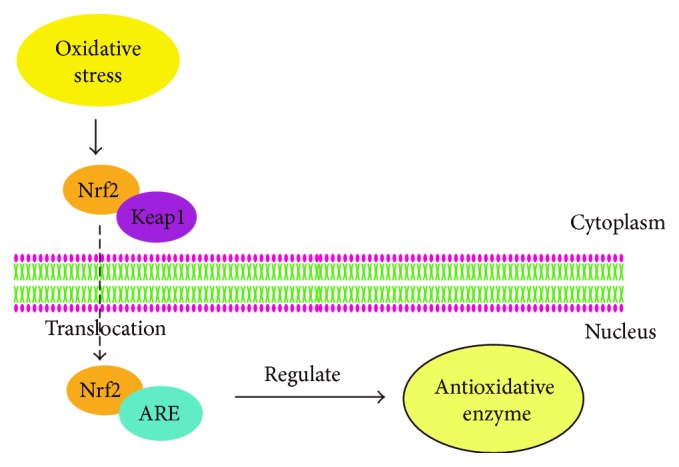
Schematic diagram of Nrf2/ARE pathway. The → indicates activation or induction. Under oxidative stress conditions, Nrf2 is separated from Keap1 in the cytoplasm and after linking with ARE is transferred to the nucleus. Then, the expression level and the amount of antioxidative enzymes regulated by Nrf2 increases and the damage caused by the imbalance of the redox potential are attenuated.

Nuclear factor-E2-related factor 2 (Nrf2) is a transcription factor, which is located in the cytosol under homeostatic conditions. Nrf2 binds to the Kelch-like ECH-associated protein 1 (Keap1) which regulates ubiquitination and degradation of Nrf2 [16]. Nrf2 modulates the expression of phase II cytoprotective enzymes, such as heme oxygenase-1 (HO-1) and *γ*-glutamylcysteine ligase (*γ*-GCL), and then increases intracellular antioxidant GSH levels [17]. On normal redox conditions, the regulation by Nrf2 leads to the production of only basal levels of cytoprotective enzymes. Traditionally, the ubiquitination and degradation of Nrf2 are carried out by Keap1 through Cullin3-based E3 ligase complex [18] and then is degraded by the proteasome. However, under oxidative stress, signals are transduced by modifying the sulfhydryl groups of the reactive cysteine in Keap1, which attenuates both polyubiquitination and proteasomal degradation of Nrf2 [19]. Meanwhile, the free Nrf2 in the cytoplasm is transferred to the nucleus to combine with antioxidant response elements (AREs), thereby increasing the level of Nrf2 in the nucleus rapidly [20]. Subsequently, the expression and amount of these antioxidative enzymes increase and attenuate the damage caused by the imbalance of the redox potential. The Nrf2/ARE pathway favors the survival response of an organism by scavenging ROS and free radical species, controlling cell cycle regulatory proteins involved in DNA damage response (DDR) and inhibiting the production of inflammatory cytokines via multiple pathways [21] (Figure 1).

Recently, Wang found that ginsenosides attenuated adriamycin-induced cardiotoxicity through improving endothelial dysfunction induced by oxidative stress, partially via the activation of the Nrf2/ARE pathway [22]. Furthermore, their study also evidenced that ginsenoside Rg3 suppressed adriamycin-induced cardiotoxicity by attenuation of endothelial dysfunction. As a result, vascular dysfunction was reversed, and cell viability and antioxidant activity increased, leading to an inhibition of apoptosis. Furthermore, the regulation of the Nrf2/ARE pathway and the expression of AKT residues were shown to be involved in these protective cellular responses.

### 6.2. NF-*κ*B Pathway

**Figure 2:**
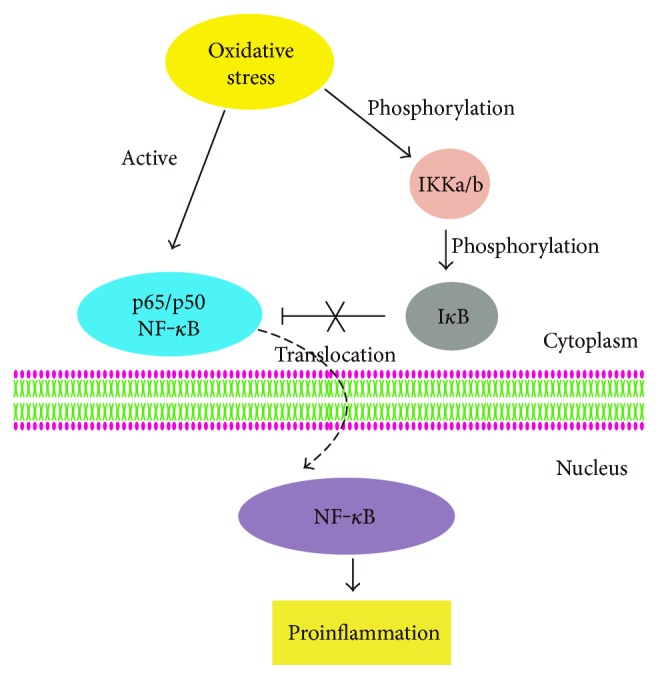
Schematic diagram of NF-*κ*B pathway. The → indicates activation or induction, and the “⊢” indicates inhibition. Under oxidative stress conditions, p65/p50 NF-*κ*B is activated. Meanwhile, I*κ*B kinase promotes the phosphorylation of IκB, causing I*κ*B to separate from NF-*κ*B. Then, NF-*κ*B is translocated from the cytoplasm to the nucleus, where it is involved in the regulation of cell proliferation and survival.

Activation of the nuclear factor kappa B (NF-*κ*B) signaling pathway has been demonstrated in endothelial cells under pathological conditions, such as atherosclerosis, diabetes, and cancer. Indeed, NF-*κ*B acts as an important transcription factor in these diseases. Naturally occurring in the cytoplasm, inactivated NF-*κ*B consists mainly of two forms, p50 NF-*κ*B and p65 NF-*κ*B, which bind to their inhibitor (I*κ*B). When oxidation of endothelial cells occurs, IκB kinase, including IKKa and IKKb, promotes phosphorylation and degradation of I*κ*B, causing I*κ*B to separate from NF-*κ*B [23]. NF-*κ*B is then translocated from the cytoplasm to the nucleus, where it takes part in the control of cell proliferation and survival. NF-*κ*B modulates the expression and suppression of the apoptosis-associated enzyme-like caspase 3, acting as a pro- or antiapoptotic factor in endothelial cells (Figure 2).

The exposure of cells to silver nanoparticles (AgNPs) has been reported to induce intracellular ROS generation, leading to cell apoptosis, activation of IKK/NF-*κ*B pathways, and, subsequently, dysfunction of cells [24, 25]. Shi et al. [26] demonstrated that AgNPs significantly increased the phosphorylation of IKKa/b and of I*κ*Ba. Furthermore, p65 nuclear translocation was also elevated after the AgNP exposure, as well increasing NF-*κ*B activity in HUVECs. Similarly, the inhibitor of oxidation N-acetyl-L-cysteine (NAC) effectively downregulated the levels of I*κ*Ba and IKKa/b phosphorylation, decreased p65 nuclear translocation, and inhibited NF-*κ*B activity. Thus, this suggests that ROS participates in endothelial cell dysfunction in AgNP-exposed HUVECs through the IKK/NF-*κ*B pathway.

NF-*κ*B can promote apoptosis in endothelial cells when endothelium is subjected to irradiation such as ultraviolet B (UVB) and hadron; this action is based on the upregulation of tumor necrosis factor alpha (TNF-*α*) and is significantly enhanced upon costimulation with interleukin-1 (IL-1) and IL-6 [27]. Apart from UVB radiation, NF-*κ*B is also commonly activated by oxidants such as H_2_O_2_. Furthermore, NF-*κ*B can act as either a pro- or antiapoptotic factor depending upon physiological conditions and cell type. In a recently published study, UVB exposure was found to augment the levels of both NF-*κ*B and pNF-*κ*B, while having a potent proapoptotic effect on HUVECs. The findings of Olteanu also indicated that doses of *Calluna vulgaris* (Cv) extract both with or without irradiation increased levels of NF-*κ*B and pNF-*κ*B, but only high doses of Cv caused oxidative stress. Oxidative stress led to the activation and expression of NF-*κ*B, thereby increasing apoptosis through increasing caspase-3 activity [28].

### 6.3. PI3K/AKT and AKT/eNOS Pathway

**Figure 3:**
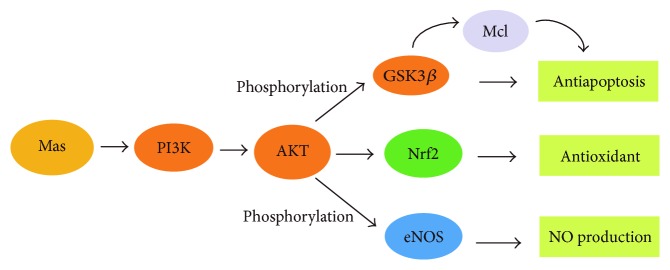
Schematic diagram of PI3K/AKT pathway. The → indicates activation or induction. In blood vessels, Mas promotes a variety of beneficial cardiovascular outcomes via the activation of the PI3K/AKT signaling pathway. Activation of the PI3K/AKT signaling pathway promotes the phosphorylation of GSK3*β* and inhibits apoptosis through increasing the expression of Mcl. Besides, the PI3K/AKT pathway plays an antioxidant role by increasing Nrf2 protein levels in the nucleus. Moreover, eNOS phosphorylation is regulated by PI3K/AKT to initiate NO synthesis.

Aging, hypertension, hyperglycemia, and a variety of other pathologies resulting from oxidative stress of endothelial cells are mediated by the PI3K/AKT signaling pathway [29]. PI3K/AKT pathway can regulate cell apoptosis and NO production and indirectly modulate the level of antioxidative enzymes. Indeed, the activation of AKT, downstream of PI3K, is considered one of the most crucial factors for cell survival (Figure 3).

Conversely, when cells are injured, p53 can act as a tumor suppressor by promoting apoptosis. Under oxidative stress, the activation of the PI3K/AKT pathway results in an increase in p53 levels, while the myc proto-oncogene protein is elevated, promoting cell senescence. In a recent study, inhibition of the PI3K/AKT pathway has been established to partly reverse the elevation of c-myc protein levels induced by angiotensin II and maintain it at a proper level. PI3K/AKT signaling pathway takes part in cell senescence and apoptosis by inducing p53, c-myc, and other important antiaging and antioxidative damage pathway proteins and survival factors [30]. As well as these important cellular factors, the antiapoptosis gene *Bcl-xl* protects endothelial cells by elevating the level of phospho-AKT [31]. GSK3*β* is a protein serine/threonine kinase which is inhibited by phosphorylation at Ser9 and is linked to the activation of PI3K and AKT [32]. A study conducted by Zhu et al. investigated whether isoquercitrin inhibits cell apoptosis via the PI3K/AKT/GSK3*β* (glycogen synthase kinase3*β*) signaling pathway. The authors revealed [33] that isoquercitrin significantly increased the expression of p-AKT and p-GSK3*β* in a dose-dependent manner in EA.hy926 cells. Furthermore, GSK3*β* regulates the phosphorylation and degradation of the proapoptotic protein Mcl. However, isoquercitrin induced GSK3*β* phosphorylation and increased the level of Mcl-1 expression, an antiapoptotic protein, through activation of PI3K/AKT signaling pathway, thus inhibiting apoptosis.

Angiotensin II has also been shown to induce endothelial dysfunction by inhibiting the expression of PI3K and phosphorylation of AKT. The PI3K/AKT/eNOS signaling pathway is downstream of Mas, a G protein-coupled receptor (GPCR). In blood vessels, Mas promotes a variety of beneficial cardiovascular outcomes via the activation of the PI3K/AKT signaling pathway. The PI3K/AKT signaling pathway mediates the eNOS activity by stimulating phosphorylation of Ser1177 residue [34], thus modulating the production of NO and subsequent endothelial-mediated vascular relaxation. Wei et al. [35] found that baicalin exerted its antioxidative effect partly through the upregulation of the PI3K/AKT/eNOS pathway, suggesting that this signaling pathway may take part in the mechanisms of the protective effects induced by baicalin.

Resveratrol (RSV) exerts protective effects on endothelial cells viability through activation of the PI3K/AKT pathway. Sui et al. [36] discovered that RSV inhibited the expression of miR-126 in HUVECs. However, the protective effects of RSV on HUVECs from H_2_O_2_-induced apoptosis were reversed when miR-126 expression was suppressed. Conversely, the overexpression of miR-126 decreased the p85-*β* expression and increased AKT phosphorylation, which regulates the activity of PI3K.

Phosphorylation of AKT may increase Nrf2 protein levels in the nucleus, and the activation of the PI3K/AKT signaling pathway can also result in an increase of HO-1 [37]. Zhuang et al. [38] found that tanshinone IIA increased AKT phosphorylation in vascular endothelial cells, and the inhibition of the PI3K pathway blocked tanshinone IIA-induced HO-1 expression. It has been demonstrated that tanshinone IIA activates Nrf2/HO-1 signaling through the PI3K/AKT pathway. The upregulation of HO-1 results from the activation of the PI3K/AKT signaling pathway and the increase in Nrf2 nuclear translocation. Furthermore, the AKT/eNOS signaling pathway mediates protection against vascular structure damage, enhanced endothelium-independent vascular function, and inhibits the abnormal proliferation of smooth muscle cells.

eNOS phosphorylation is regulated by PI3K/AKT to initiate NO synthesis. AKT activation-mediated eNOS phosphorylation governs NO release. AKT also plays a critical role in the control of phosphorylation of eNOS-ser1177 in vivo. Li et al. [39] revealed that the upregulation of phosphorylated AKT and eNOS induced by fucoidan was counteracted by the suppression of AKT and eNOS phosphorylation protein expression. PI3K and AKT are downstream effectors of insulin signaling and are important signaling molecules in the regulation of glycogen metabolism in myocytes, lipocytes, and hepatocytes.

By regulating angiogenesis, proliferation, microvascular permeability, survival, cellular transformation, and embryonic differentiation, PI3K/AKT also exerts an essential influence in the regulation of endothelial cell (EC) function. Through PI3K/AKT signaling, cells respond to a variety of cytokines, G protein-coupled receptor ligands, and growth factors [40], as well as to cellular stresses, including heat shock, hypoxia, and oxidation.

High glucose significantly reduces the phosphorylation of AKT and GSK3*β*. Uncoupling of insulin signaling at PI3K-AKT in response to high glucose concentrations in these cell types has been implicated in the pathogenesis of insulin resistance. The phosphorylation and inactivation of GSK3*β*, a downstream effector of AKT, are considered important mechanisms of cell survival. Wang et al. confirmed that decreased GSK3*β* phosphorylation levels are responsible for high-glucose-induced oxidative damage, a finding that was even more prevalent under intermittent high-glucose conditions. The modified phosphorylation levels of GSK3β are attributed to the increase in the phosphorylation of AKT. Pretreatment of HUVECs with *Panax quinquefolius* saponin (PQS) significantly reversed decreased GSK3*β* phosphorylation levels induced by the intermittent high-glucose treatment through PI3K/AKT/GSK3*β* pathway [41, 42].

### 6.4. AMPK Pathway

**Figure 4:**
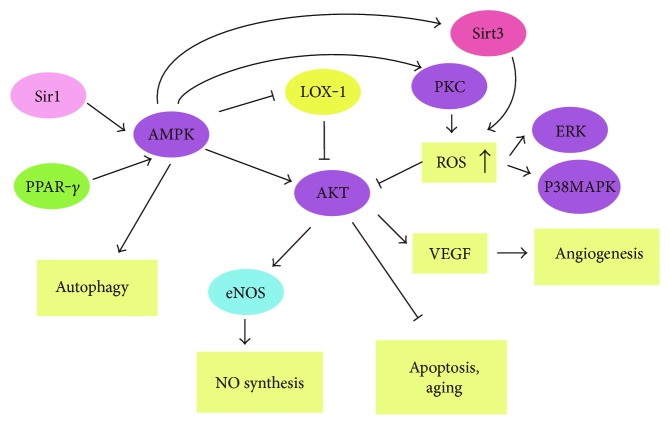
Schematic diagram of AMPK pathway. The → indicates activation or induction, and the ⊢ represents inhibition. Sirt1 has been reported to contribute to AMPK activation. Sirt1 and AMPK act upstream of the autophagy regulation pathway. PPARc activates and moderates the activation of AMPK, causing a decrease in the expression of LOX-1. It is noteworthy that LOX-1 is essential for ox-LDL-mediated inhibition of AKT and eNOS activity and downstream NO synthesis. The AKT phosphorylation increased by AMPK activation mediates the VEGF-dependent angiogenesis and decreases apoptosis and aging. Moreover, AMPK signaling pathway participates in the oxidative stress via the combined effect of ERK and p38MAPK on the regulation of ROS production.

In endothelial cells, AMP-activated protein kinase (AMPK) suppresses oxidative stress-induced injury in endothelial cells by maintaining cellular energy balance and metabolism [43]. Apart from regulating cell growth and metabolism, AMPK activation also induces autophagy. Indeed, dysregulation of AMPK signaling pathway has been implicated in oxidative stress [44]. AMPK has several isoforms, including AMPK-*α*1, *β*1, and *γ*1 that regulate multiple signal transduction pathways, exerting vascular protective effects, such as decreasing intracellular ROS formation, attenuating NADPH oxidase (NOX) activation, activating the AKT pathway, enhancing NO synthesis, and reducing the adhesion of inflammatory cells to the endothelium of blood vessels (Figure 4).

Protein kinase C (PKC) is downstream of AMPK. Inhibition of PKC expression contributes to the attenuation of NOX-derived ROS production. The increased amount of endothelial ROS is a major factor for the development of endothelial dysfunction and cardiovascular disease. Furthermore, NOX-activated ROS functions as a second messenger molecule to initiate downstream signal conduction pathways, such as activation of p38MAPK, stimulation of NO catabolism as a result of superoxide generation, and suppression of NO release through attenuated eNOS phosphorylation, thereby activating NF-*κ*B and triggering downstream proinflammatory responses [45].

Ox-LDL decreases the level of phosphorylated AMPK. It is noteworthy that oxidative stress-induced LOX-1 (ox-LDL receptor) is essential for ox-LDL-mediated inhibition of AKT and eNOS activity and downstream NO synthesis. PPARc-mediated AMPK activation controls the expression of LOX-1. Ciglitazone, a peroxisome proliferator-activated receptor c (PPARc), mediates PPARc activation and moderates the activation of AMPK, causing a decrease in the expression of LOX-1. In addition, AMPK activation increases AKT phosphorylation. Ciglitazone, a drug used to treat diabetes, mediates the VEGF-dependent angiogenesis, endothelium-dependent relaxation, and protection of RMVECs against ageing and apoptosis. This antihyperglycemic agent promotes NO synthesis through activation of AKT and upregulation of eNOS expression and therefore contributes to the protection of mitochondrial homeostasis in endothelial function, with PPARc-dependent AMPK activation which is critically involved in the process of macrophage proliferation, and increased cellular cholesteryl esters are suppressed by ciglitazone. In an earlier investigation, PPARc ligands have been shown to significantly suppress high-glucose-mediated ICAM-1 expression. LOX-1 is regulated by PPARc at the posttranscriptional level; thus, AMPK-PPARc signaling feedback may protect endothelial cells against atherosclerosis through mediating cellular oxidative stress stimulated by the ox-LDL/LOX-1 axis [3].

In addition, some agents, such as Sirtuin 1 (Sirt1), a mammalian ortholog of yeast silent information regulator 2 (Sir2), protect endothelial cells from oxidation by AMPK and other regulatory factors. Hung et al. [46] found that quercetin mitigated oxLDL-induced oxidative injury in HUVECs. Quercetin also upregulated Sirt1 expression and increased AMPK activity, thus providing protective effects against oxidative damage. The signaling pathways of quercetin-mediated antiatherosclerotic effects can reduce ROS formation and maintain mitochondrial function. Zhou et al. [47] demonstrated that RSV phosphorylates and activates AMPK, which is required for PGC-1*α* expression. PGC-1*α* then activates ERR*α* to cause ERR*α* binding to ERRE in the mating-type information regulation 2 homolog 3 (Sirt3) promoter regions, leading to silent Sirt3 mRNA transcription. The AMPK/PGC-1*α*/Sirt3 signaling pathway may be a key for the regulation of mitochondrial ROS homeostasis by RSV treatment in endothelial cells [48].

Autophagy is an important catabolic process through which cytoplasmic components are delivered to the lysosome for degradation. Impaired or excessive autophagy leads to an accumulation of damaged proteins and intracellular organelles and promotes cell death. Generally, autophagy is a cell protective mechanism, but, depending on the cellular conditions, may also enhance cell death. Hyperglycemia impairs autophagy in HUVECs through inhibition of the phosphorylation of AMPK, activating the downstream effector mTOR through mTOR phosphorylation and leading to endothelial cell damage. Liang et al. demonstrated that ampelopsin, which functions by targeting autophagy via AMPK activation, had a protective effect against hyperglycemia-induced cell damage. During oxidative stress in endothelial cells, ampelopsin initiated an autophagic survival response via the AMPK/mTOR signaling pathway. This process, advanced by activation of AMPK phosphorylation, including the phosphorylation of TSC2, Raptor, or ULK1, resulted in the inhibition of mTOR and the promotion of autophagy in a dose-dependent manner. AMPK-dependent autophagy induction has been intensively identified as a protective mechanism against primary diabetic complications occurring in cardiomyocytes and mesangial cells [49]. Autophagy plays an important role in the protective effect on oxidative damage in endothelial cells. Sirt1 and AMPK have been shown to act upstream of the autophagy regulation pathway. In addition, the activation of AMPK is possibly increased in the cellular AMP/ATP ratio [43]. Sirt1 is activated downstream, most likely from AMPK activation increasing the NAD^+^/NADH ratio, and Sirt1 has been identified as NAD^+^-dependent. It is noteworthy that Sirt1 has been reported to contribute to AMPK activation through deacetylation of the upstream kinase, LKB1. In in vitro experiments, RSV activates AMPK suggesting that RSV activates Sirt1 indirectly. This finding revealed the crucial role of the regulation of autophagy through Sirt1/AMPK signal pathway in the protective effects of RSV [50].

AMPK plays a key role in multiple signaling transduction pathways, including cell proliferation, cell death, and migration. Zhou et al. [51] found that high-glucose-induced oxidative stress was related to suppression of the phosphorylation status of AMPK*α*. RSV ameliorated high-glucose-induced damage in HUVECs in a time-dependent manner by activation of AMPK*α*, led to enhanced cellular reductive reactions, and mediated oxidative stress. Moreover, their results support the hypotheses that AMPK inactivation contributes to oxidative stress damage, and AMPK reactivation is restorative to cellular processes.

In conclusion, oxidative stress in endothelial cells is a complex process consisting of many contributing factors. There are a plethora of mechanisms associated with a variety of signaling pathways that require further study. Moreover, drugs and other exogenous stimuli have a complicated influence on endothelial cells, perhaps acting through several pathways. The findings on the signaling pathways described in this article are forefront, and the mechanisms involved are still largely unclear. This review provides several schematic diagrams and clearly shows certain possible patterns of oxidative stress within endothelial cells. The conclusions presented herein provide a necessary basis for further investigation within the field to better understand the actions of a variety of stimuli on endothelial cells and to elucidate the beneficial effects of treatment with various therapeutic agents in the prevention of cardiovascular disease.
